# A Case, Illustrative of the Beneficial Effects of Cold Water, as an External Application

**Published:** 1839-10-19

**Authors:** C. A. Finley

**Affiliations:** Surgeon U. S. Army; Fort Monroe


To the Editors of the Medical Examiner.

*A Case, illustrative of the Beneficial Effects of Cold Water, as an External Application.* By C. A. Finley, M. D., Surgeon U. S. Army.

To the Editors of the Medical Examiner.

Gentlemen:—Having observed in your interesting journal a report of the beneficial effect of the application of cold, by the syphon and cold water, in lacerated wounds, I am induced to lay before you the history of a case, further illustrative of the antiphlogistic and sedative effect of cold water, which came under my charge in the course of my service.

On the 4th of July, 1831, —— Van Alstein, a private of the fifth regiment of infantry, was brought to my hospital, severely wounded by the premature discharge of a cannon, whilst in the act of ramming home the charge. On examination, I found a compound comminuted fracture of both bones of the fore-arm, with extensive laceration of the soft parts, precluding the possibility of saving the limb. He was a man of intemperate habits, and partially intoxicated at the time the accident occurred. I amputated above the elbow joint, and, the weather being extremely warm, made the dressing as light as possible. On my morning visit, I was told he had passed a very uncomfortable night, complaining much of the jerking of the stump, which was hot and tumid as high as the shoulder; the spasms were so violent as to raise it from the pillow. I enveloped the stump in a fold of patent lint, and having ordered a basin of water, with ice in it, an attendant was placed by his side, who was instructed to keep, by means of a sponge, a continued gentle stream of the cold water pouring on the lint. Under this treatment, in a short time, the puffiness, spasms, and every unfavourable symptom, disappeared, and the stump healed by the first intention, with the exception of the angle through which the ligatures passed.

Would not this treatment be highly prophylactic where traumatic tetanus is expected?

*Fort Monroe*, October 9, 1839.

